# Zoonotic spillover: Understanding basic aspects for better prevention

**DOI:** 10.1590/1678-4685-GMB-2020-0355

**Published:** 2021-06-04

**Authors:** Joel Henrique Ellwanger, José Artur Bogo Chies

**Affiliations:** 1 Universidade Federal do Rio Grande do Sul, Departamento de Genética, Programa de Pós-Graduação em Genética e Biologia Molecular, Laboratório de Imunobiologia e Imunogenética, Porto Alegre, RS, Brazil.

**Keywords:** Zoonosis, spillover, pathogen, infectious diseases, COVID-19

## Abstract

The transmission of pathogens from wild animals to humans is called “zoonotic spillover”. Most human infectious diseases (60-75%) are derived from pathogens that originally circulated in non-human animal species. This demonstrates that spillover has a fundamental role in the emergence of new human infectious diseases. Understanding the factors that facilitate the transmission of pathogens from wild animals to humans is essential to establish strategies focused on the reduction of the frequency of spillover events. In this context, this article describes the basic aspects of zoonotic spillover and the main factors involved in spillover events, considering the role of the inter-species interactions, phylogenetic distance between host species, environmental drivers, and specific characteristics of the pathogens, animals, and humans. As an example, the factors involved in the emergence of Severe Acute Respiratory Syndrome Coronavirus 2 (SARS-CoV-2) pandemic are discussed, indicating what can be learned from this public health emergency, and what can be applied to the Brazilian scenario. Finally, this article discusses actions to prevent or reduce the frequency of zoonotic spillover events.

## Introduction

Outbreaks and epidemics are frequent events in human history. Some of them take on pandemic proportions, reaching populations on different continents. As a matter of fact, several pathogens that infect humans are derived from other animal species. Conservative estimates indicate that around 60% of all emerging human infectious diseases have a zoonotic origin (Woolhouse and Gowtage-Sequeria, 2005; Jones *et al.*, 2008). Other estimates suggest that this percentage reaches up to 75% (Taylor *et al.*, 2001). Regardless of the differences between these estimates, it is evident that most human infectious diseases have a zoonotic origin. In other words, these diseases originate from pathogens that were transmitted from non-human animals to humans, through a process known as zoonotic spillover. For instance, HIV was introduced in the human population from non-human primates in the 1920s or even before, and the AIDS pandemic emerged between the 1960s and 1990s, subsequently rapidly spreading throughout the world (Gilbert *et al.*, 2007; Faria *et al.*, 2014; Worobey *et al.*, 2016; Gryseels *et al.*, 2020).

The Severe Acute Respiratory Syndrome Coronavirus (SARS-CoV) emerged in humans in 2002/2003 and the Middle East Respiratory Syndrome Coronavirus (MERS-CoV) in 2012. Both these coronaviruses were likely derived from viral strains found in bats. Palm civets and dromedary camels potentially acted as intermediate hosts for the introduction of the SARS-CoV and MERS-CoV in the human population, respectively (de Wit *et al.*, 2016). In late 2019, a new respiratory disease called Coronavirus Disease 19 (COVID-19) emerged in China. The disease is caused by the Severe Acute Respiratory Syndrome Coronavirus 2 (SARS-CoV-2), an RNA virus belonging to the *Coronaviridae* family, genus Betacoronavirus. This new human coronavirus has spread rapidly between different countries, causing the COVID-19 pandemic (Wu *et al.*, 2020).

Activities and factors that increase the interaction of humans with different animal species and pathogens they host, which include handling, poaching, and consumption of meat from wild animals and derived products, are associated with increased risk of spillover events (Kurpiers *et al.*, 2016; Ellwanger *et al.*, 2020). For example, the HIV spillover from non-human primates to humans occurred through the manipulation of meat of primates probably slaughtered for human consumption (Hahn *et al.*, 2000). In addition to serving as a source of food, in many countries, wild animals and their products are also sold in live animal markets (“wet markets”) for medicinal purposes or cultural practices, as souvenirs, pets, among other finalities. These markets contribute significantly to the interaction of humans with different species and new pathogens. In those places where different species are confined, pathogens can be transmitted not only through contact with meat, blood and other biofluids, but also through aerosols and contaminated surfaces (Brown, 2004; Lo *et al.*, 2019; Aguirre *et al.*, 2020; Wassenaar and Zou, 2020). Activities in sylvatic environments, such as deforestation and construction of human settlements in forest areas, also increase the interaction between humans and different species and for this reason are important drivers of spillover events. The accelerated growth of the world population and the increased loss of global biodiversity, as a result of human activity, suggest that spillover events will become more and more frequent (Ellwanger *et al.*, 2020).

It is necessary to understand which factors facilitate spillover events, in order to apply actions to reduce the frequency of transmission of pathogens from wild animals to humans, minimizing the risk of new outbreaks, epidemics, and pandemics. In this context, the first part of this review describes the basic aspects of zoonotic spillover and the main factors involved in spillover events, considering characteristics of hosts (humans and other animal species), environment, and pathogens. Also, the factors and the potential animal species involved in the emergence of the SARS-CoV-2 pandemic are discussed, aiming to consolidate lessons that can be applied to the Brazilian scenario. Finally, this article describes actions to prevent or reduce the frequency of zoonotic spillover events. In sum, the objective of this article is to review the basic aspects of zoonotic spillover from a One Health perspective, covering the several aspects associated to the environment, humans, pathogens, and non-human animals.

## Methodological notes

This article is structured as a narrative review. The manuscript was written based on searches in the databases PubMed/MEDLINE (https://pubmed.ncbi.nlm.nih.gov/), SciELO (https://scielo.org/) and Google Scholar (https://scholar.google.com.br/), using the terms “spillover”, “zoonotic spillover”, “pathogen spillover”, “host jump”, “cross-species transmission” and “zoonotic transfer”, considering articles published in English and without restriction for year of publication. The articles that described basic and conceptual aspects regarding zoonotic spillover were included in this review. In addition to original articles, theoretical works describing discussions or concepts relevant to the topics covered in the review were also included. From this initial selection of key papers, we complemented the article with discussions on specific topics based on the selection of papers in a more specific way, with targeted searches using terms related to topics of interest (e.g., MERS-CoV, HIV, SARS-CoV-2, COVID-19), and also using the electronic library on zoonotic spillover and related content maintained by the authors in recent years. Of note, although this article was written during the COVID-19 pandemic, we bring also examples unrelated to SARS-CoV-2/COVID-19 since the topics discussed here apply not only to the current pandemic, but also to outbreaks, epidemics and pandemics occurred in the past and those that will certainly happen in the future, on a smaller or larger scale. This review was written with the aim of being accessible to a wide audience of readers, from different research fields. For that reason, basic and introductory aspects of zoonotic spillover are mentioned. This is not an exhaustive review of the topic.

### Basic aspects of zoonotic spillover

The transmission of pathogens between different species, the crossing of species barriers, is an ecological phenomenon known as “host jump”, “cross-species transmission”, “zoonotic transfer”, “pathogen spillover”, or “zoonotic spillover” (Lu *et al.*, 2015; Plowright *et al.*, 2017; Knetsch *et al.*, 2018; Becker *et al.*, 2019). Specifically, “spillover” can be defined as the “cross-species transmission of a parasite into a host population not previously infected” (Wells and Clark, 2019). Usually, spillover refers to the cross-species transmission of pathogens from wildlife (vertebrate animals) to humans (Plowright *et al.*, 2017; Wells and Clark, 2019). The transmission of a pathogen from humans to wildlife (reverse zoonosis), by direct contact between species or mediated by vectors, can be called “spillback” (Weaver, 2013; Hendy *et al.*, 2020; Olival *et al.*, 2020). Finally, “horizontal virus transfer” is a term that can be used to describe the transmission (regardless of direction) of viruses between different species, including viral transmission between organisms of different biological kingdoms (Dolja and Koonin, 2018).

Spillover is a complex and multifactorial phenomenon, involving aspects associated with the hosts, microorganisms and the environment. The risks of a spillover event occurring will be primarily influenced by the prevalence and intensity of infection in reservoir hosts, usually non-human animals from wild environments or farms. The distribution and density of infected hosts in a given environment is also a determining factor for the spillover risk (Plowright *et al.*, 2017). The prevalence and intensity of infection in reservoir hosts are relevant because these factors determine the pathogen load in such species and the patterns of pathogen shedding. Both factors are influenced by the immunological conditions of the hosts, the interactions between different species (e.g., prey-predator interactions, habitat sharing) and environmental aspects (e.g., characteristics of ecosystem boundaries, presence/absence of environmental degradation). The pathogen load observed in reservoir hosts influences the amount of pathogens that will be excreted in the environment (e.g., through feces) or will be found in the meat and biofluids of these animals. Of note, the characteristics of pathogens also influence the risk of spillover events since they determine the viability of the pathogen in the environment and its ability to be transmitted between different hosts or vectors (Plowright *et al.*, 2017; Becker *et al.*, 2019; Borremans *et al.*, 2019). 

**Figure 1 -:**
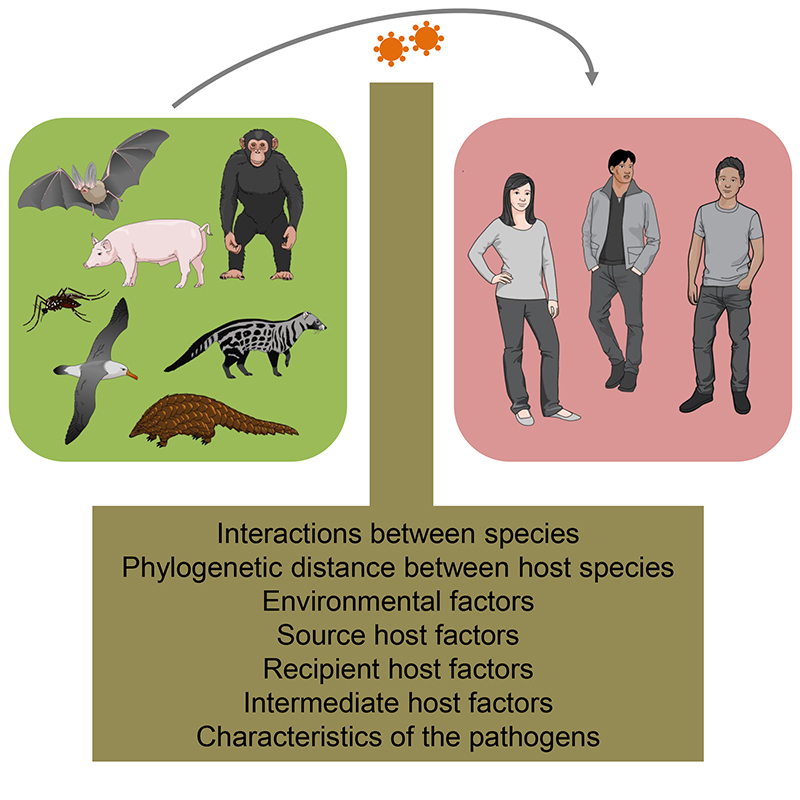
Factors between wildlife, livestock animals and humans. The intensity and frequency of interactions between species, the phylogenetic distance between host species, characteristics of the pathogens, source host-associated factors, recipient host-associated factors, environmental factors, and intermediate host-associated factors (when this particular host is present) can hinder or facilitate the transmission of pathogens from wild animals/livestock to the human population. The zoonotic spillover occurs when these factors are favorable to the pathogen, allowing the crossing of barriers between species. This figure was created using *Mind the Graph* illustrations (available at www.mindthegraph.com).

Ecological and intrinsic factors related to the microorganisms influence the risk of spillover events even before the interaction with humans, since other animals and pathogens could also be involved in the process. Then, the frequency of human contact with animals as well as the dose and route of exposure to the pathogens will modulate the risk of spillover. Looking specifically at human related factors, biological (e.g., genetics, immune status, physical state of skin and mucous membranes) and social aspects (e.g., cultural practices, eating habits, housing characteristics) will affect the mode, intensity and frequency of interactions with different host species or vectors (Plowright *et al.*, 2017; Becker *et al.*, 2019; Borremans *et al.*, 2019). Indeed, there are several barriers and factors between wildlife organisms and humans that hinder the transmission of pathogens from wild animals to the human population (Figure 1). The same applies to pathogens transmitted from livestock animals to humans. These barriers must be overcomed in zoonotic spillovers, and will be discussed in the next section.

**Figure 2 -:**
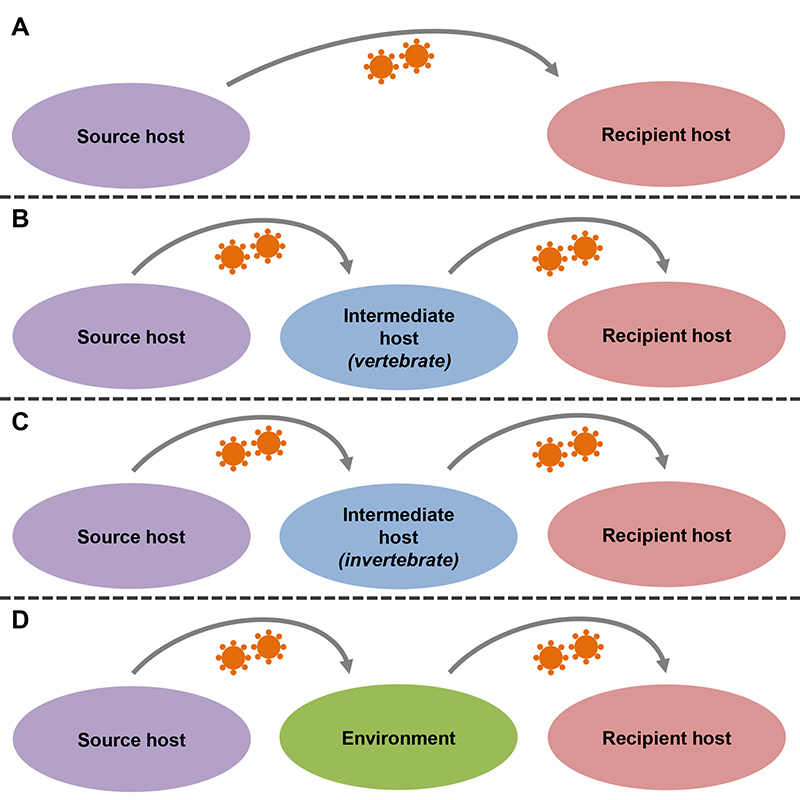
Models of zoonotic spillover. Pathogens can overcome the barriers between species in different ways. A: the pathogen can be transmitted directly from the source host to the recipient host (e.g., a virus is transmitted from a bat to a human, via a bat bite). B: the pathogen can be transmitted from the source host to an intermediate vertebrate host and then be transmitted to the recipient host (e.g., a protozoan is transmitted from a wild species to a domestic dog, which then transmits the parasite to a human host). C: the pathogen can be transmitted from the source host to an intermediate invertebrate host and then be transmitted to the recipient host (e.g., an arbovirus is transmitted from a wild primate to a mosquito, which then transmits the virus to a human host). D: the pathogen can be transmitted from the source host to the environment and then be transmitted to the recipient host (e.g., a swine species releases an enterovirus into the environment through feces, which subsequently infects a human who has come into contact with the animal’s feces present in the environment).

Spillover generally involves (I) a “source host”: species responsible for shedding the pathogen; (II) a “recipient host”: a species that is infected by the pathogen from a different host; and (III) a “bridge/intermediate host”: a host species that acts as a bridge or link in the transfer of the pathogen between species. The intermediate host can be a vertebrate host or an invertebrate vector, such as a mosquito. The intermediate host may or may not be present in the spillover event (Borremans *et al.*, 2019). Many pathogens survive outside the host without losing viability and transmissibility, and therefore the environment can act as an intermediary in the transmission of pathogens between different species. In other words, exposure of the recipient host to the new pathogen can occur either directly (e.g., through an animal bite or contact with contaminated secretion/blood) or indirectly (e.g., through a vector, or contact with contaminated feces or a surface containing the microorganism) (Wolfe *et al.*, 2005; Kurpiers *et al.*, 2016; Borremans *et al.*, 2019). Figure 2 shows schematically the main ways that new pathogens can be introduced in a human population. 

**Figure 3 -:**
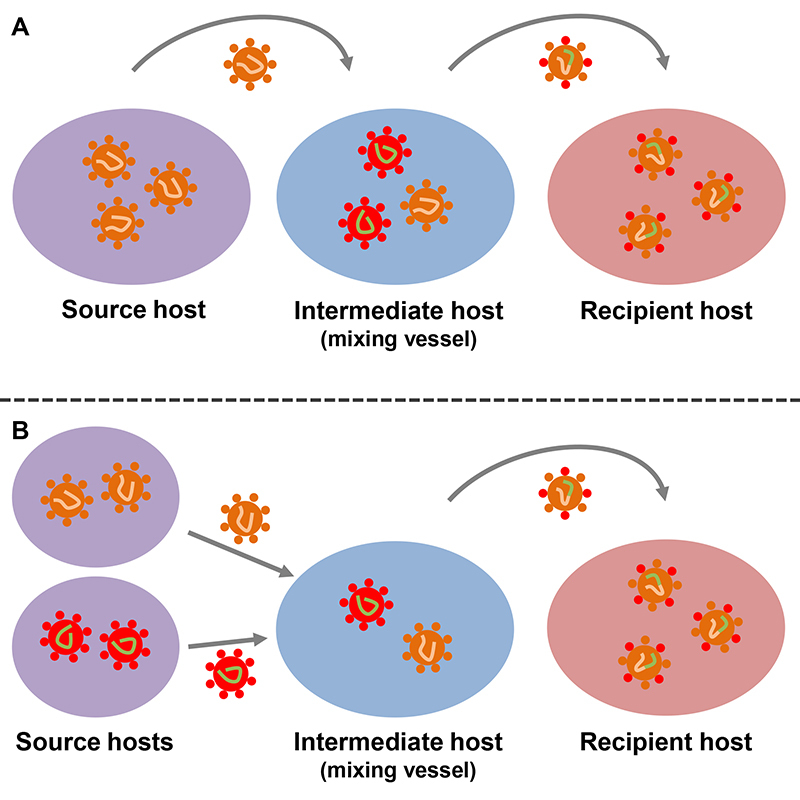
Intermediate host as a “mixing vessel” for the emergence of new viral strains. When an intermediate vertebrate host is infected by different viral strains, recombination or reassortment can occur between viral species, generating new viral strains. The intermediate host can function as a mixing vessel of new viral trains in two ways. A: a viral strain from a source host exchanges genetic information with a viral strain originally present in the intermediate host, producing a new viral strain. B: the intermediate host is infected with viral strain from two (or more) source hosts, allowing the exchange of genetic information between the viral species, producing a new viral strain. Hypothetical viral recombination in an intermediate host is exemplified in this figure, but the same processes can be applied to viral reassortment.

Intermediate hosts can also serve as a “mixing vessel” for the emergence of new viral strains when such a host is infected by two or more viruses (Figure 3). In brief, a new (hybrid) viral strain from parental viruses can originate from recombination or reassortment. In recombination, gene fragments from parental viruses are recombined, resulting in a virus with a chimeric genomic molecule that contains regions of nucleotide sequence derived from each parental virus. In reassortment, two or more parental viruses exchange genome segments, also originating a hybrid virus (McDonald *et al.*, 2016). For example, swine species can host influenza viruses from birds and humans, allowing genetic reassortment and the emergence of new viral strains (Shi *et al.*, 2014).

**Figure 4 -:**
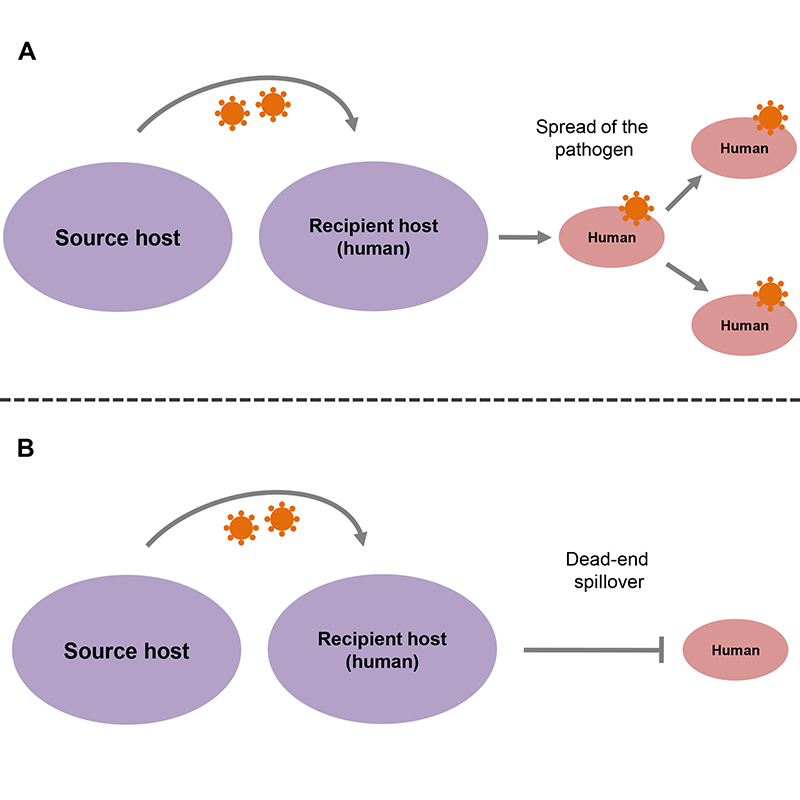
Outcomes after a spillover event. A spillover event can have two basic outcomes. A: the pathogen that crossed the barriers between species can spread among the population (through human-to-human transmission or a highly competent vector). This outcome will occur if the social, biological, and environmental conditions are favorable for the adaptation of the pathogen in the human host and its transmission among the new population. B: the pathogen is transmitted to a human, but without spreading among the human population due to the absence of social, biological, or environmental conditions favorable to the adaptation of the pathogen to the human population or its dissemination. This condition is called dead-end spillover.

The intensity and frequency of human interactions with livestock, companion animals, and wild species are very large. Such interactions create even more opportunities for the occurrence of spillover events. However, not all spillover events result in new human diseases or epidemics. The emergence of a new human disease and its dissemination in the population occurs only when biological, social, and environmental conditions are favorable for the replication/adaptation of the pathogen in the human host and its transmission among the new population. In general, if the pathogen shows low virulence or if its transmissibility is limited, the spillover will be of no medical importance. In other words, there will be no new human disease or the pathogen will not spread among the population. “Dead-end spillover” is an expression generally used to characterize the situation when a pathogen is transmitted to a human, but this event is not followed by spreading among the human population. Although spillover events that result in new human diseases are not common, when they do occur, the impacts can be very important, either by the appearance of a new human disease *per se*, or by the spread of the new disease on a large scale (Wolfe *et al.*, 2005; Morse *et al.*, 2012; Geoghegan *et al.*, 2016; Plowright *et al.*, 2017). Figure 4 shows schematically a dead-end spillover and a spillover followed by the spread of the pathogen among the population. 

Finally, it is important to emphasize that the transmission of microorganisms between different species, without human-associated disease, is common in nature. For example, it is currently known that inter-kingdom horizontal virus transfer is a frequent phenomenon and has broadly influenced the evolution of RNA viruses (Dolja and Koonin, 2018).

## Factors that influence zoonotic spillover

### Interactions between species

A basic condition for the occurrence of a spillover event is the interaction between different species. In this sense, the chances of new pathogens being introduced in the human population increase as the interactions between humans and other animal species are more intense and frequent. Therefore, as expected, geographic range overlap facilitates the transmission of pathogens between species (Albery *et al.*, 2020).

The industrial meat production chain might put the human population away from the risks associated with contact with farm animals and their pathogens. On the other hand, the growing demand for meat production puts meat industry workers, veterinarians and farmers in close and growing contact with livestock animals, such as poultry, swine, and cattle. Adding to this, sometimes professionals of the meat industry do not have adequate working conditions to reduce the risks of infection by zoonotic pathogens or do not know sufficiently, or neglect, the health risks involved in their work activities (Cunha *et al.*, 2012; Tebug *et al.*, 2015; Hundal *et al.*, 2016; Klous *et al.*, 2016). Livestock animals are sources of different human pathogens, such as influenza virus strains, bacteria, and protozoa (Tomley and Shirley, 2009). Companion animals can also transmit zoonotic diseases/pathogens to humans, including parasites, bacterial and viral diseases, and fungal infections. Rabies is a classic example of viral zoonosis often transmitted from unvaccinated domestic animals to humans, especially in low and medium income countries (Chomel, 2014). 

The interaction of humans with wild species plays a prominent role in the emergence of new human diseases since wild animals are natural reservoirs of many unknown pathogens. As previously mentioned, HIV emerged in the human population from the interaction with meat/blood of wild primates (Hahn *et al.*, 2000; Sharp and Hahn, 2011). Also, Nipah, Ebola and Hendra are examples of pathogens that caused outbreaks in the human population from the interaction of humans with other animal species, which is frequently associated with changes in the behavior of wild species resulted from anthropogenic modifications of the natural environment (Fiel *et al.*, 2001; Pigott *et al.*, 2014; Saéz *et al.*, 2015; Clayton, 2017). Sociocultural factors such as hunting, trade and consumption of meat from wild species (bushmeat) facilitate spillover events because these human actions put humans in close contact with fresh meat, offal and blood of animals that can host different pathogens (Hahn *et al.*, 2000; Wolfe *et al.*, 2005). As a general rule, the interaction between humans and animal species, in different ways, is a fundamental step for a spillover event. However, factors specific to pathogens, humans and animals will define whether the human contact with a new pathogen will or will not result in disease and, eventually, in an outbreak or epidemic. Therefore, to understand the aspects that determine zoonotic spillover events, it is necessary to consider the One Health approach, integrating human, animal and environmental factors in the study, surveillance and prevention of emerging zoonotic diseases (Thompson, 2013; Bonilla-Aldana *et al.*, 2020; Marty and Jones, 2020).

### Phylogenetic distance between host species

The phylogenetic distance between source and recipient hosts is an important factor in spillover events. It is assumed that the chances of a pathogen being transmitted between phylogenetically similar species are greater compared to the chances of transmission between phylogenetically distant hosts (Davies and Pedersen, 2008; Han *et al.*, 2016; Olival *et al.*, 2017). For instance, the spillover of pathogens between two species of mammals (non-human primates to humans, for example) would be a more likely event than a spillover event involving a species of mammal and a reptile. However, this is not a stricter rule. The spillover of a particular pathogen between phylogenetically distant species may occur if there is interaction between these species and the pathogen finds basic conditions of infection and adaptation in the new host (Parrish *et al.*, 2008; Walker *et al.*, 2018; Albery *et al.*, 2020).

Source and recipient hosts with high genetic similarity also show similarities regarding body physiology and cellular receptors (Walker *et al.*, 2018). As a consequence, since similarity increases the chance of the virus interact with cells of the new host, high similarity of cellular receptors between host species can facilitate the spillover of viral pathogens (Bae and Son, 2011; Cho and Son, 2019; Wasik *et al.*, 2019). Moreover, host genetic factors dictate the ability of a virus to replicate in a new host and, therefore, to establish productive infection and transmission in the new population (Warren and Sawyer, 2019).

Recently, Guth *et al.* (2019) evaluated how phylogenetic distance impacts the virulence and transmissibility of zoonotic viruses. In brief, the authors analyzed primarily a database containing 420 virus-mammal associations including 67 zoonotic viruses and 278 mammalian hosts, and found some patterns involving transmissibility and virulence. First, Guth *et al.* (2019) corroborate that the risk of spillover is higher among species with greater phylogenetic proximity. The authors also observed that reservoir animals phylogenetically close to humans generally host zoonotic pathogens of lower impact associated with morbidity and mortality (reduced virulence), but with greater transmissibility between humans. On the other hand, animals with a greater phylogenetic distance from humans are more likely to host highly virulent zoonoses (viruses with increased virulence), but with a limited capacity for human-to-human spread (reduced transmissibility) (Guth *et al.*, 2019).

Comparing the mammalian orders Primates, Perissodactyla, Carnivora, Cetartiodactyla, Chiroptera, Eulipotyphla, Lagomorpha and Rodentia, bat-borne viruses (viruses from Chiroptera) were classified as the most virulent agents of mammalian zoonoses (Guth *et al.*, 2019). However, bats can also host viruses with low or moderate virulence (e.g., coronaviruses) and, therefore, these particular bat-borne viruses can indeed spread widely among the human population in some situations, as observed in the SARS-CoV-2 pandemic.

### Characteristics of the pathogens

Generalist pathogens, with the ability to infect a broad host range, are more able to jump the barrier between species than specialist pathogens (Johnson *et al.*, 2015). Some characteristics of the pathogens facilitate the transmission between species. For example, viruses with an RNA genome have higher mutation rates and more frequent gene rearrangements. These characteristics increase the chances of adaptation to new host species (Nichol *et al.*, 2000). Indeed, most zoonotic viruses have RNA genome (Johnson *et al.*, 2015). Moreover, some authors found an association between viral replication in the cytoplasm and greater zoonotic potential (Pulliam and Dushof, 2009; Olival *et al.*, 2017).

Different viral taxonomic groups have varied zoonotic potential (Olival *et al.*, 2017; Washburne *et al.*, 2018). For example, looking at mammalian viruses, the *Alphavirus* and *Deltaretrovirus* genera have a large proportion of zoonotic viral species. On the other hand, the families *Papillomaviridae*, *Herpesviridae*, *Caliciviridae*, *Adenoviridae*, and *Astroviridae* have few zoonotic representatives. This demonstrates that distinct taxons can be associated with varied risks of spillover. Based on zoonotic representatives and viral characteristics, the family *Togaviridae* has a high spillover potential. On the other hand, the family *Herpesviridae* shows low spillover potential (Washburne *et al.*, 2018).

In the case of spillover involving indirect transmission, the resistance of the pathogen in the environment or feces/secretions of animals will determine the success of the transmission between species. Non-enveloped viruses are more resistant outside the host, a feature that facilitates indirect transmission (Geoghegan *et al.*, 2016; Walker *et al.*, 2018).

After a spillover event, the ability of a viral species to be transmitted by direct contact (human-human transmission) facilitates its dissemination among the human population. The ability to cause long-term chronic infection, the presence of non-segmented genome, the absence of viral envelope, high host plasticity, and the infection of the respiratory tract are some characteristics that can facilitate the spread of viral species, favoring the emergence of an outbreak or epidemic (Johnson *et al.*, 2015; Geoghegan *et al.*, 2016; Walker *et al.*, 2018; Guth *et al.*, 2019). However, it is important to highlight that some viral characteristics, such as the capacity of human-human transmission and chronic infection, are most strongly associated with the ability of a pathogen to spread among the population after a spillover event, and do not necessarily facilitate spillover events *per se*, although some specific viral characteristics, such as the virus stability in the environment, may facilitate both processes (Geoghegan *et al.*, 2016; Walker *et al.*, 2018).

Finally, the extensive and often inappropriate use of antimicrobial drugs in the medical and veterinary field, especially antibiotics, acts in the selection of microorganisms resistant to different classes of such drugs (Woolhouse *et al.*, 2015). For instance, data from Brazilian hospitals indicate that the rate of methicillin-resistant *Staphylococcus aureus* ranges from 30% to 60%, and may be above 60% in some locations (Rossi, 2011). Microbial resistance is also a growing problem in livestock animals of Brazilian farms (Rabello *et al.*, 2020). As a consequence, treatment options for multiresistant strains become increasingly scarce and create new opportunities for the circulation of resistance genes between humans, other animals and the environment (Woolhouse *et al.*, 2015). 

### Animal host-associated factors

Animal species act as natural reservoirs for microbial species, eventually serving as “sources” of new human pathogens. Specifically, wild species are reported as the source of most zoonotic viral diseases (Johnson *et al.*, 2015). For example, bats are classically known as natural reservoirs of various pathogens, especially viruses, which cause disease in humans (Calisher *et al.*, 2006). The role of bats as reservoirs of multiple viruses is due to the capacity of the immune system of these animals to deal with a wide variety of pathogens. Although it was suggested that this particular immune function may be favored by the increased body temperature and metabolism observed in bats (O’Shea *et al.*, 2014), the immune mechanisms that enable bats to deal with different viruses are still under investigation (Ahn *et al.*, 2019).

The ecological habits of bats, which frequently share the environment with humans and domestic/livestock animals (Gomes *et al.*, 2010; Esbérard *et al.*, 2014; Russo and Ancillotto, 2015; Nunes *et al.*, 2017), the abundance and high diversity of bat species in nature (Mickleburgh *et al.*, 2002; Calisher *et al.*, 2006), and the abundance of zoonotic pathogens found in such animals (Olival *et al.*, 2017) are some factors that can facilitate the spillover of pathogens from bats to humans. 

Bats have already been involved in the emergence of different human infectious diseases, such as Ebola, Nipah, Hendra, and SARS (Saéz *et al.*, 2015; Wang and Anderson, 2019). Bats have received special attention in recent years among researchers investigating potential new human pathogens in wildlife. This focus on bats has likely overestimated their role in the emergence of infectious diseases. Recent data indicate that other animal groups can host as many, or even more, zoonotic pathogens as bats. Of note, there is little variation in proportion of zoonotic viruses found in different taxonomic orders (Mollentze and Streicker, 2020). Besides bats, other species may also play an important role in the emergence of new human infectious diseases but are being neglected, including wild rodents and livestock animals for meat production. 

The production of meat and derived products cause direct impacts (livestock as source of human pathogens) and indirect effects (e.g., contributing to biodiversity loss and climate change) on the dynamics of infectious diseases (Rohr *et al.*, 2019; Morand, 2020). Livestock animals can transmit pathogens to humans, contributing to the emergence of epidemics and pandemics. For example, different influenza strains, such as H1N1pdm09, were introduced into the human population after viral reassortment in swine reared for human consumption (Shi *et al.*, 2014). According to Cleaveland *et al.* (2001), 77% of pathogens from livestock animals can infect multiple hosts. Swine species can act as reservoirs of many other pathogens, including hepatitis E virus, *Streptococcus suis*, and *Taenia solium* (Da Silva *et al.*, 2018; Chu *et al.*, 2019). Livestock and domestic animals can also transmit pathogens to wild species (Mahapatra *et al.*, 2015; Bevins *et al.*, 2018). Conversely, wild animals can transfer pathogens to livestock (Musoke *et al.*, 2015). 

Along with livestock and wild species, domestic animals can act as source host of many human pathogens, including emerging viruses (Reperant *et al.*, 2016). For example, cat-to-human transmission of the avian influenza A virus (H7N2) was recently reported (Lee *et al.*, 2017; Poirot *et al.*, 2019).

In addition to serving as natural reservoirs of pathogens, animals may have other roles in the transfer of pathogens between different species. Some animals can act as bridges/intermediate hosts in spillover events. For example, dogs can easily transit between domestic environments and forest areas, potentially transferring pathogens from wild animals to humans. This phenomenon occurs especially in urban areas located close to forest landscapes (Ellwanger and Chies, 2019). Animal vectors can also act as intermediate hosts. In this sense, mosquitoes can carry arboviruses from wild primates to humans, being at the interface of the sylvatic and urban cycles of arboviral diseases such as Dengue and Yellow Fever (Chen and Vasilakis, 2011; Weaver, 2013; Possas *et al.*, 2018).

### Environmental factors

The loss of biodiversity is associated with the emergence and spread of infectious diseases. Conversely, forests and other natural landscapes with high abundance of animal species have a greater capacity to “maintain” pathogens in the wild environment, reducing the risk of zoonotic spillover from wildlife to humans (LoGiudice *et al.*, 2003; Keesing *et al.*, 2010; Civitello *et al.*, 2015; Ostfeld, 2017; Gibb *et al.*, 2020). In this sense, LoGiudice *et al.* (2003) showed that the preservation of vertebrate biodiversity was associated with reduced incidence of Lyme disease. An explanation for the pivotal role of biodiversity on health promotion is the “dilution effect”. In brief, high diversity of host species decreases, or “dilutes”, the prevalence of infection in high competent reservoir hosts, which reduces the risk of spillover events and human infection (Schmidt and Ostfeld, 2001; Johnson and Thieltges, 2010; Civitello *et al.*, 2015; Khalil *et al.*, 2016; Ostfeld, 2017). Also, biodiverse landscapes have many species that interfere with the transmission of pathogens through numerous mechanisms, reducing the risk of human infection (Johnson and Thieltges, 2010; Civitello *et al.*, 2015).

Land-use changes reduce the richness and abundance of terrestrial species (Newbold *et al.*, 2015) and are one of the main drivers of emerging infectious diseases, facilitating the emergence of vector-borne and zoonotic diseases (Loh *et al.*, 2015; Gibb *et al.*, 2020). In brief, extensive agriculture and the transformation of forest areas in pasture facilitate the emergence and spread of new human diseases since these processes favor the proliferation of generalist small species that host many pathogens (e.g., rodents), increase the load of pathogens in such species, and also contribute to climate change, which affects vector dynamics (e.g., expansion of areas infested by mosquitoes that transmit arboviruses). In sum, the risk of zoonotic diseases increases in places where natural landscapes are replaced by croplands, pastures or have been submitted to other human-related land changes (Keesing *et al.*, 2010; Loh *et al.*, 2015; Rohr *et al.*, 2019; Wilke *et al.*, 2019; Ellwanger *et al.*, 2020; Gibb *et al.*, 2020; Mendoza *et al.*, 2020; Ostfeld and Keesing, 2020). 

In Brazil, land-use changes and agricultural intensification are among the main drivers of the emergence of zoonotic diseases in humans, considering data from 1940 to 2005 (Keesing *et al.*, 2010). Also, environmental degradation and loss of biodiversity promote the spread of animal vectors and pathogens to different areas, facilitating the transposition of pathogens between different species. In this sense, the construction of roads, human habitations and hydroelectric power plants in forest areas, in addition to other deforestation-associated activities, approximates humans to wild species and their pathogens, creating the basic conditions for the occurrence of zoonotic transfer (Ellwanger *et al.*, 2020). In accordance, Ebola outbreaks in Africa are associated with forest fragmentation and deforestation (Rulli *et al.*, 2017). Although the reasons for this association are not completely clear, anthropogenic changes in forest areas probably put humans in closer contact with Ebola reservoirs due to ecological and social factors linked to modifications in reservoir behavior and disturbances to wildlife, including hunting, bushmeat consumption, and landscape changes (Rulli *et al.*, 2017). Moreover, land-use changes and environmental conditions that facilitate the proliferation of animal vectors and urban rodents can facilitate the introduction of pathogens in the human population and the spread of diseases (Himsworth *et al.*, 2013; Cruvinel *et al.*, 2020).

Several environment-related factors (e.g., abiotic conditions, parasite pressure, the presence of contaminants) affect the immune system of different species and populations, including humans, livestock and wild animals. Therefore, environment-related factors affect the immune system of source hosts, influencing the abundance and diversity of pathogens found in such animals, as well as the immune defenses of recipient hosts. As a consequence, the risks of spillover events will also be dependent on the effects of the environment on the immune system of source and recipient hosts. The immunological peculiarities of source species distributed across different geographic regions will also affect the risk of spillover events, which adds complexity to the understanding of disease ecology (Davis, 1998; Bowden, 2008; Bean *et al.*, 2013; Kreitinger *et al.*, 2016; Becker *et al.*, 2020).

Finally, environmental factors such as microclimate and rain patterns modify the behavior of reservoir species and recipient hosts, as well as the viability of the pathogens in the environment. As a consequence, these modifications affect the occurrence of spillover events and the risk of transmission of pathogens from wildlife to humans (Martin *et al.*, 2017, 2018; Schmidt *et al.*, 2017; Cortes *et al.*, 2018; Giles *et al.*, 2018).

### Human host-associated factors

Many factors that influence spillover events are modulated by human activity, including land-use change, interactions with different animal species, and the trade and consumption of meat from wild animals. In addition to these sociocultural factors, immunological and genetic factors also affect the human susceptibility to infections.

The characteristics of the immune responses (e.g., activity of Toll-like receptors and Th1/Th2 cell types) dictate the abundance and variety of pathogens found in the host, since the immune system modulates the susceptibility to infections and the adaptation of a particular pathogen to the host (Mukherjee *et al.*, 2016; Ellwanger *et al.*, 2018a). For instance, immunocompromised individuals are more susceptible to infection by opportunistic pathogens (Kovacs and Masur, 2000; Brown *et al.*, 2012). Also, susceptibility/resistance to infectious diseases is strongly modulated by host genetic factors (e.g., HLA alleles and chemokine genes), including polymorphisms in coding and non-coding regions (Cooke and Hill, 2001; Quintana-Murci *et al.*, 2017; Ellwanger *et al.*, 2018b). For example, the *NLRP3* (rs10754558) gene polymorphism was associated with protection against HTLV-1 infection in individuals from northeastern Brazil (Kamada *et al.*, 2014). Recently, a study involving HIV-infected individuals showed that the *IL-4* (-590C/T, rs2243250) gene polymorphism is associated with susceptibility to *Pneumocystis jirovecii* pneumonia (Wójtowicz *et al.*, 2019). From a broader perspective, these data indicate that the immune status and genetic factors influence the susceptibility/resistance of humans to new infectious diseases from wildlife, thus affecting the chances of a new pathogen adapting to the human population (Matzaraki *et al.*, 2017; Plowright *et al.*, 2017; Ellwanger *et al.*, 2018b).

Finally, it is important to highlight that humans can transmit pathogens to non-human hosts (“reverse zoonosis”), potentially introducing diseases in new animal populations (Nelson and Vincent, 2015). For instance, humans can transmit *Mycobacterium tuberculosis* to cattle (Mittal *et al.*, 2014). Strains of the influenza virus are often transmitted from humans to swine (Nelson and Vincent, 2015). Also, a probable human-to-porcine transmission of rotavirus strains was recently reported in Brazil (Neves *et al.*, 2020), and there is evidence of the transmission of SARS-CoV-2 from humans to dogs (Sit *et al.*, 2020). Reverse zoonosis is a concern in terms of both public health and wildlife conservation (Olival *et al.*, 2020).

#### Zoonotic spillover and emerging infectious diseases: looking at Chinese and Brazilian contexts

In late 2019, cases of an unknown infectious respiratory disease were detected in the city of Wuhan, Hubei province, central China. Early investigations pointed out that many individuals affected by the new disease had frequented a popular wet market in Wuhan, where various species of animals were sold for human consumption, as well as animal products used in traditional Chinese medicine. This information raised concerns of scientists and health officials, who realized that a new disease from wild species was emerging in the human population (Cohen and Normile, 2020; Cyranoski, 2020a, 2020b).

A few weeks after the first cases of infection were reported, the genomic material of the etiologic agent of this new disease was sequenced and characterized. The pathogen was identified as a Betacoronavirus, the SARS-CoV-2, an RNA virus that causes the disease now known as COVID-19 (Cohen and Normile, 2020; Liu, 2020; Wu *et al.*, 2020). Currently, the COVID-19 pandemic is already the most important public health emergency of recent human history. 

Some aspects of this pandemic have been compared to the 1918-1919 Spanish Flu, the “mother of all pandemics” (Taubenberger and Morens, 2006), due to the medical, economic, and social impacts that SARS-CoV-2 has unleashed on society. For example, Spanish Flu and COVID-19 show similar case-fatality rates (~3%) (Taubenberger and Morens, 2006; Lee, 2020). However, these data should still be taken with caution, since the COVID-19 pandemic is ongoing and the actual case-fatality rate can change. Interestingly, due to the current high number of commercial air travel, the COVID-19 pandemic spread around the world much faster than the Spanish Flu (Lee, 2020). Also, it is likely that the number of deaths from COVID-19 will be much lower than that recorded in the Spanish Flu due to the particular characteristics of SARS-CoV-2 and the current global health systems. Spanish Flu affected approximately 500 million individuals worldwide, causing ~50 million deaths (Taubenberger and Morens, 2006). About a year after the COVID-19 pandemic emerged, SARS-CoV-2 infected more than 69 million individuals worldwide and caused approximately 1.6 million deaths (data collected on December 10, 2020). On the same date, China registered more than 93 thousand cases and Brazil was close to reaching 8 million cases (Dong *et al.*, 2020; Johns Hopkins University, 2020). The number of SARS-CoV-2-infected individuals is probably much higher than the official reports because most cases of infection are mild or asymptomatic (Zhang and Holmes, 2020). Looking at human-related biological aspects, SARS-CoV-2 has spread easily around the world because the *ACE2* gene receptor (which encodes the protein used by the virus to penetrate cells) is highly conserved in different human populations (Fam *et al.*, 2020).

Recent genomic analyzes show that SARS-CoV-2 is similar to coronaviruses found in bats: 96% similar to BatCoVRaTG13 strain (Zhou *et al.*, 2020a) and 93% similar to RmYN02 strain at level of complete virus genome (Zhou *et al.*, 2020b). However, these bat viral strains are sufficiently different from SARS-CoV-2 to indicate the existence of an intermediate host. Bats are source hosts of the MERS-CoV and SARS-CoV, other coronaviruses responsible for human epidemics in past recent years (de Wit *et al.*, 2016). Taking together, these findings and others (Latinne *et al.*, 2020) suggest that bats are also involved in the emergence of the COVID-19 pandemic, as a host source, and that another animal could have acted as an intermediate host. 

Human SARS-CoV pandemic occurred between 2002 and 2003, causing 774 deaths in 27 countries. MERS-CoV emerged in the human population in 2012, causing more than 600 deaths and also affecting 27 countries (de Wit *et al.*, 2016). In SARS-CoV and MERS-CoV outbreaks, palm civets (*Paguma larvata*) and dromedary camels (*Camelus dromedarius*), respectively, acted as intermediate hosts during zoonotic spillover. In other words, these animals facilitated the transmission of SARS-CoV/MERS-CoV precursor coronaviruses from bats to humans (Guan *et al.*, 2003; Chu *et al.*, 2014; de Wit *et al.*, 2016; Jin *et al.*, 2020; Rodriguez-Morales *et al.*, 2020). In the case of SARS-CoV, it is probable that, besides palm civets, other species acted as intermediate hosts (de Wit *et al.*, 2016), including raccoon dogs (*Nyctereutes procyonoides*) (Guan *et al.*, 2003; Mallapaty, 2020).

Much debate exists about which species would have acted as an intermediate host in the emergence of SARS-CoV-2 in the human population. Genomic analysis indicates that pangolins (*Manis javanica*) may have played this role, once these animals host coronaviruses very similar, genetically, to SARS-CoV-2 (Lam *et al.*, 2020; Zhang *et al.*, 2020). Of note, these animals are used in traditional medicine and are appreciated in the Chinese gastronomic culture, moving a large trade of these animals and raising great concerns in terms of conservation, especially because pangolins have a low reproductive rate (one offspring per year) (Liu and Weng, 2014; Zhou *et al.*, 2014). On the other hand, some data support that the viral strain that originated SARS-CoV-2 may have been circulating in bat populations for many years (maybe since the 1940s or before that) and subsequently been transmitted to humans without necessarily the participation of an intermediary host (Boni *et al.*, 2020). Indeed, although the initial genomic analysis indicated that pangolins may have been intermediate hosts in the SARS-CoV-2 spillover, other animals can also host coronavirus strains as similar to SARS-CoV-2. These other animals may just have not been evaluated yet. In other words, in addition to the probable involvement of bats as natural reservoirs of SARS-CoV-2-related coronaviruses, the data already accumulated do not allow us to state which animal species were indeed involved in the introduction of SARS-CoV-2 in the human population (Zhang and Holmes, 2020). The World Health Organization intends to investigate in the field the conditions that contributed to the emergence of SARS-CoV-2 in the human population. The investigation will cover the city where the fist cases of COVID-19 were detected, Wuhan, as well as other Chinese cities and countries (Mallapaty, 2020).

It is also not possible to state the particular steps that SARS-CoV-2 took until the introduction and spread in the human population. The introduction of a pathogen from a wild animal to the human population can be a gradual (multistep) process, through several spillover events, as well as involving multiple intermediate hosts (Wolfe *et al.*, 2007; Bean *et al.*, 2013; de Wit *et al.*, 2016). Also, SARS-CoV-2 may have surged from recombination of coronaviruses of bats and pangolins. In this case, pangolins would have acted as an intermediate reservoir and a “mixing vessel” for the emergence of SARS-CoV-2 (Li *et al.*, 2020; Xiao *et al.*, 2020). However, these assumptions remain under investigation and much debate (Boni *et al.*, 2020), and the pieces of this puzzle continue to be assembled.

On the other hand, the available data is already sufficient to affirm that SARS-CoV-2 is a virus resulting from natural processes involving the interaction of humans with other species (Andersen *et al.*, 2020). In China, wet markets are part of the national culture (Zhong *et al.*, 2020), demanding large volumes of wild and live animals to supply restaurants and family consumption. It was estimated that 2 to 30 tons of live wild animals are transported into China daily (Yang *et al.*, 2000; Wang *et al.*, 2020). Inspection failures associated with poor sanitary conditions in wet markets create ideal conditions for spillover events in such places. This is the likely scenario in which the COVID-19 emergence occurred in the human population (Zhang and Holmes, 2020). However, the trade and consumption of wild animals and derived products are not exclusive to China but are present in several countries, including Brazil. Hunting and commercialization of meat from wild animals are intense and poorly inspected activities in Brazil, where several wild species are used for food and medicinal purposes (Alves *et al.*, 2012; Sanches *et al.*, 2012; Fonseca and Pezzuti, 2013; van Vliet *et al.*, 2014; Chagas *et al.*, 2015; Torres *et al.*, 2016; Souto *et al.*, 2018; El Bizri *et al.*, 2020). However, there are many particularities in the demand for wild animals in Brazil and China.

Few data are available on the volume of wild meat commercialized in Brazil. In the Amazonian trifrontier of Brazil, Colombia and Peru, more than 470 tons of meat from wild animals is commercialized per year, as estimated by van Vliet *et al.* (2014). However, it is likely that this volume is even greater because such trade is illegal in such Amazonia countries and, therefore, very difficult to measure (van Vliet *et al.*, 2014). An older study estimated that up to 89,224 tons of wild meat is consumed each year in the rural Brazilian Amazon (Peres, 2000). Of note, van Vliet *et al.* (2014) also estimated the commercialization of wild meat at 3.2 kg/habitant/year for the studied region (Brazil-Colombia-Peru trifrontier). A recent study estimated that 10,691 tons of wild meat are consumed annually in 62 cities of central Amazon. This amount of wild meat represents 6.49kg/habitant/year (El Bizri *et al.*, 2020).

Brazil is one of the world’s largest producers of cattle meat (Neto, 2018). The availability of cattle meat in Brazil certainly reduces the consumption of bushmeat since it creates greater food security concerning the availability of this type of protein, at least at the national level. On the other hand, a study in the Brazil-Colombia-Peru trifrontier identified the average price of fresh bushmeat in the market places as USD$ 5.32/kg (van Vliet *et al.* 2014). Since the price of bushmeat is comparable to the price of fresh beef (USD$6.2/kg), this can serve as a stimulus for bushmeat consumption where this type of meat is available and part of the local culture. Another study performed in the central Brazilian Amazon estimated the price of one kg of wild mammal or bird for fresh meat at ~USD$2.00 (and even less for dried meat) (Chaves *et al.*, 2019), indicating that in some places of Brazil the consumption of meat from wild animals can indeed be financially advantageous.

As a consequence of the scenario mentioned above, the possibility of the emergence of new human infectious diseases in Brazilian territory exists and is generally neglected. However, it is necessary to draw the parallel between China and Brazil with criticism. The trade and consumption of meat from wild animals and derived products is part of Chinese culture and occurs in a huge scale. In Brazil, this is an illegal practice and is limited to some specific regions and populations, on a small scale compared to China. Besides, Brazil has a limited sales volume of wild meat (usually associated with illegal trade) compared to the widespread Chinese wet markets.

Previously the SARS-CoV-2 emergence, many researchers had warned that a new epidemic caused by a coronavirus could occur, causing an important impact on human society. They highlighted the need to improve surveillance and enhance the development of antiviral drugs and vaccines focused on coronaviruses (de Wit *et al.*, 2016; Devi, 2017; Ng and Tan, 2017; Rabaan, 2017). In 2018 we call attention to the need to intensify the surveillance of zoonotic diseases and spillover events in the Brazilian territory (Ellwanger and Chies, 2018). These alerts must be translated into a more robust inspection of illegal hunting activities and commercialization of meat from wild animals in Brazil, associated with the intensification of epidemiological surveillance focusing on human-animal interfaces (Ellwanger *et al.*, 2019). For example, many spillover events and limited outbreaks involving the Ebola virus in Africa are not detected by health systems or international health authorities (Glennon *et al.*, 2019). Similarly, in Brazil, cases of several infectious diseases remain underreported due to the lack of compulsory notification of some diseases, misdiagnosis, and underdiagnosis (Dias *et al.*, 2011; Oliveira *et al.*, 2013; Brito and Teixeira, 2017; Rodrigues, 2017; Shikanai-Yasuda *et al.*, 2017; Almeida *et al.*, 2019). Hospital-based surveillance of zoonotic exposures can be very useful for early detection of the emergence of infectious diseases, in addition to providing more detailed information on spillover events (Das *et al.*, 2019). These lessons should be taken very seriously in Brazil once the country is a hotspot for the emergence of new infectious diseases due to its rich diversity of animal species that host many zoonotic diseases and potential new human pathogens (Hotez, 2014; Han *et al.*, 2016; Olival *et al.*, 2017; Ellwanger and Chies, 2018). This is of particular concern in Brazil because in the country there is a growing anthropogenic interference in natural landscapes, such as the Amazon rainforest (Ellwanger *et al.*, 2020). Brazil is also considered a hotspot for the emergence of zoonotic diseases because it concentrates different activities associated with land-use changes (e.g., agricultural practices) in the Amazon region (Ellwanger *et al.*, 2020). As previously mentioned, land-use changes are among the main drivers of emerging infectious diseases (Keesing *et al.*, 2010; Loh *et al.*, 2015; Gibb *et al.*, 2020).

In addition to the environmental problems observed in the Amazon region, Brazil harbors other highly biodiverse biomes. The Atlantic Forest, a huge biodiversity hotspot, has been degraded since the beginning of the European colonization in the 1500s, resulting in intense forest fragmentation and loss of habitats and diversity of plant and animal species (Ribeiro *et al.*, 2011; Maioli *et al.*, 2020). To the best of our knowledge, there are no estimates of the number of new zoonotic diseases that have emerged in the human population as a result of the degradation of this biome. However, some examples can be mentioned. In the 1970s, Brazil faced an important epidemic of human encephalitis caused by the emergence of the mosquito-borne Rocio virus in *Vale do Ribeira* and *Baixada Santista*, both regions of Atlantic Forest in the state of São Paulo (Lopes *et al.*, 1978; Iversson, 1980). More recently, Brasil *et al.* (2017) reported an outbreak of *Plasmodium simium*-associated human malaria in the Atlantic Forest of Rio de Janeiro state. *Plasmodium simium* is traditionally considered a monkey-specific malaria parasite, and such data demonstrate the zoonotic transmission of the parasite in the region of the Atlantic Forest (Brasil *et al.*, 2017). Finally, the 2016-2018 yellow fever outbreak affected wild primates and humans in Atlantic Forest regions of southeastern Brazil, which had remained free of yellow fever outbreaks for more than 80 years (Abreu *et al.*, 2019).

Several other pathogens circulate in mosquito vectors (Barrio-Nuevo *et al.*, 2020; Ximenes *et al.*, 2020), bats (de Araujo *et al.*, 2012; Góes *et al.*, 2016; Gonçalves-Oliveira *et al.*, 2020), wild rodents (Rozental *et al.*, 2017; Vieira *et al.*, 2019), non-human primates (Costa *et al.*, 2014; Catenacci *et al.*, 2018) and dogs (Curi *et al.*, 2014; Sevá *et al.*, 2018) found in portions of the Atlantic Forest and adjacent areas in different regions of Brazil. The interaction of animals with human populations that live in close contact with the Atlantic Forest can cause isolated cases of zoonotic infections in humans, maintain the endemicity cycles of diseases such as Leptospirosis and Leishmaniasis (Curi *et al.*, 2014; Vieira *et al.*, 2019), as well as create the ideal conditions for the emergence of new human infectious diseases in Brazil.

The urbanization process that occurs in areas previously occupied by forests favors the proliferation of vectors of different diseases. Currently, Brazil has a series of endemic arboviruses circulating in the Atlantic Forest region, such as Dengue, Zika and Chikungunya. The ecological connection between urban and forest environments, in association with the occurrence of different species of mosquitoes of medical importance in Atlantic Forest areas (*Aedes albopictus*, *Aedes serratus*, *Aedes scapularis*, *Psorophora ferox*, *Haemagogus leucocelaenus*, among many others) (Cardoso *et al.*, 2011; Laporta *et al.*, 2012; Alencar *et al.*, 2016a,b), gives support for the circulation of different arboviruses between sylvatic and urban areas (Weaver, 2013; Figueiredo, 2019). Based on this scenario, it is likely that vector-mediated spillover (and spillback) events are the main problem in Brazil in terms of the risks of emergence of infectious diseases, unlike in China, where the greatest risks may be related to the bushmeat trade. However, this is an interpretation that needs to be further investigated.

Epidemiological surveillance, early case identification and blocking measures are essential to adequately respond to the emergence of a new disease. It is important to note that China identified very rapidly the virus responsible for the COVID-19 pandemic, having sequenced the pathogen’s genome in weeks after its emergence (Cohen and Normile, 2020; Liu, 2020). In situations of epidemics and pandemics, rapid responses are essential for understanding and controlling the disease. In Brazil, the SARS-CoV-2 genomes were also rapidly sequenced after the first two infection cases were detected in the country (de Jesus *et al.*, 2020), which was essential to understand the dynamics of the pandemic in Brazil in an agile way. These good examples reinforce the importance of Brazilian scientists and health officials to strengthen the national structure of genome-based surveillance of emerging pathogens, as exemplified by studies addressing Chikungunya (Nunes *et al.*, 2015), Zika (Faria *et al.*, 2017), Yellow Fever (Hill *et al.*, 2020) and SARS-CoV-2 (Candido *et al.*, 2020) in the country. A surveillance strategy using next-generation technologies will be very useful to contain diseases that may emerge in the national territory, potentially affecting other countries, or that will eventually arrive in Brazil from other places (Ellwanger *et al.*, 2017). 

There are many problems in the Brazilian health system that need to be addressed, such as the national capacity for risk communication, health emergency response, laboratory capacity, environmental sanitation (Vicente, 2020). Finally, strengthening the Brazilian Unified Health System (*Sistema Único de Saúde* - SUS) is essential for future public health emergencies to be properly managed and contained in Brazil. Similar to the essential role played by SUS in controlling past public health emergencies, such as the Zika virus epidemic, the SUS is proving essential for the treatment of most COVID-19 patients (Ventura *et al.*, 2020). The lack of engagement of a portion of government officials and population in taking effective measures to contain the spread of SARS-CoV-2 (e.g., wearing masks, social distance) is very worrying during the COVID-19 pandemic in Brazil and indicates that these aspects need be broadly improved so that the country could more effectively face future public health emergencies involving infectious diseases.

#### Preventing spillover events

Considering the factors that increase or decrease the risks of transfer of pathogens between species, together with the lessons learned from past outbreaks, epidemics, and pandemics, it is possible to list some actions to prevent spillover events based on the One Health perspective (Ellwanger and Chies, 2018; Ellwanger *et al.*, 2020). Of note, many of these actions are also useful for the control of diseases already observed in human populations, such as vector-borne infections. Put in simple, the actions are: Improve sanitary control of livestock; Increase surveillance of pathogens at human-animal interfaces; Control hunting and trade of wild animals; Reduce deforestation and biodiversity loss; Improve the infrastructure of basic/environmental sanitation; Avoid the construction of human housing in forest areas; Control vectors and free-ranging animals; Increase investments in human training and laboratories focused on the identification of new pathogens and emerging diseases as well as the development of vaccines; Identify biological and social factors of susceptibility to infections; Create financial funds to finance the mitigation of the emergence of infectious disease outbreaks, shortly after spillover events; Regulate biosafety protocols for professional working with wildlife and livestock.

Based on the history of health crises, it is known that funds and health actions focused on outbreaks and epidemics tend to disappear after the end of such public health emergencies (Ventura *et al.*, 2020). Therefore, it is necessary that actions focused on prevention of spillover events were applied constantly and systematically, preventing the emergence of new epidemics realistically. Finally, it is fundamental that the prevention and mitigation of public health emergencies took into account the social, political, and economic aspects of each population (Ventura *et al.*, 2020).

## Conclusion

Understanding the factors involved in zoonotic spillover events is essential to the development of better actions to control and prevent the emergence of new human diseases. Stimulating the study of the transmission of pathogens between species is very important, especially in Brazil, where few research groups are dedicated to this topic.

Importantly, no species should be eliminated or stigmatized due to the potential risks posed to humans. Each species has an ecological role in nature that must be respected. For example, although bats host a variety of pathogens, these animals provide key ecosystem services, such as pollination, pest control, seed dispersal, and are involved in several other ecological aspects (Mickleburgh *et al.*, 2002; Olival, 2016; Pereira *et al.*, 2020). The popular prejudice against bats is very worrying because many species of bats are at risk of extinction (Mickleburgh *et al.*, 2002).

Although bats and other animal species are frequent “sources” of new human diseases, they are not the cause of these diseases. Human activities are the main facilitators of the transfer of pathogens from non-human animals to humans. Therefore, measures to prevent emerging diseases must be focused on human activities and the way humans interact with other species.

Spillover events and the emergence of new infectious diseases are connected. New pandemics such as COVID-19 will certainly occur in the next years, to a lesser or greater extent. Although these public health emergencies are recurrent in human history and cannot be avoided completely, it is possible to minimize the risk of occurrence of such phenomena. The intensity of each pandemic will also be a reflection of the health system. Researchers and health authorities must work together with the society to identify the main risk factors for spillover events in each region or country, applying actions to prevent them and strengthen the actions to rapidly contain diseases emergence after a spillover event.

Finally, spillover and emerging infectious diseases are highly connected with the way humans interact with animal species and the environment. This connection increases society’s responsibility for the preservation of Brazilian biodiversity, once it is now very evident that nature preservation is also a public health need.
